# Graphene Oxide Quantum Dots Covalently Functionalized PVDF Membrane with Significantly-Enhanced Bactericidal and Antibiofouling Performances

**DOI:** 10.1038/srep20142

**Published:** 2016-02-02

**Authors:** Zhiping Zeng, Dingshan Yu, Ziming He, Jing Liu, Fang-Xing Xiao, Yan Zhang, Rong Wang, Dibakar Bhattacharyya, Timothy Thatt Yang Tan

**Affiliations:** 1 Singapore Membrane Technology Center, Nanyang Environment and Water Research Institute, Interdisciplinary Graduate School, Nanyang Technological University, Singapore; 2 School of Chemical and Biomedical Engineering, Nanyang Technological University, Singapore; 3 School of Chemistry and Chemical Engineering, Sun Yat-sen University, Guangzhou, China; 4 Department of Chemical and Materials Engineering, University of Kentucky, Lexington, KY, USA

## Abstract

Covalent bonding of graphene oxide quantum dots (GOQDs) onto amino modified polyvinylidene fluoride (PVDF) membrane has generated a new type of nano-carbon functionalized membrane with significantly enhanced antibacterial and antibiofouling properties. A continuous filtration test using *E. coli* containing feedwater shows that the relative flux drop over GOQDs modified PVDF is 23%, which is significantly lower than those over pristine PVDF (86%) and GO-sheet modified PVDF (62%) after 10 h of filtration. The presence of GOQD coating layer effectively inactivates *E. coli* and *S. aureus* cells, and prevents the biofilm formation on the membrane surface, producing excellent antimicrobial activity and potentially antibiofouling capability, more superior than those of previously reported two-dimensional GO sheets and one-dimensional CNTs modified membranes. The distinctive antimicrobial and antibiofouling performances could be attributed to the unique structure and uniform dispersion of GOQDs, enabling the exposure of a larger fraction of active edges and facilitating the formation of oxidation stress. Furthermore, GOQDs modified membrane possesses satisfying long-term stability and durability due to the strong covalent interaction between PVDF and GOQDs. This study opens up a new synthetic avenue in the fabrication of efficient surface-functionalized polymer membranes for potential waste water treatment and biomolecules separation.

One of the most pervasive challenges afflicting the human race is inadequate access to clean water and sanitation^1^,^2^,^3^, and this is due to the rapid technological advancement going in tandem with high population growth. A huge threat to public health worldwide is caused by pathogenic microorganisms in natural and sewage water. Membrane technologies are finding wide applications in water treatment^4^, but the growth of pathogenic microorganisms on the membrane surface can produce a fouling layer, *i.e.*, biofilm, and this leads to significant decrease in water flux and increase in energy consumption. Functionalization of water filtration membranes with microorganism resistance property is therefore important in overcoming pathogenic contamination and biofouling^5^,^6^. Poly(vinylidene fluoride) (PVDF) microfiltration and ultrafiltration membranes, widely used in industrial wastewater and municipal water treatment, possess excellent chemical and thermal stability, high resistance to radiation and strong mechanical property^7^. However, PVDF is intrinsically hydrophobic and therefore easily contaminated by microorganism, resulting in serious membrane fouling^8^. Therefore, new modification strategies of PVDF membranes for effective and long-lasting antibacterial and antibiofouling activities will be highly attractive.

Incorporating biocidal nanostructure materials into membranes has been regarded as an effective way to impart antimicrobial and antibiofouling properties to the membrane. Biocide-releasing nanomaterials, such as nano-metal oxide (zinc oxide)^9^, silver and copper nanoparticles^10^,^11^, have been introduced inside the membrane. However, these nanomaterials are often leached out during the water purification process, resulting in secondary contamination^6^. Alternatively, carbon-based nanomaterials, including one-dimensional (1 D) carbon nanotubes and two-dimensional (2 D) graphene nanosheets, have been explored as excellent antimicrobial reagents due to their cytotoxic mechanisms of physical piercing and oxidative stress^5^,^12^,^13^,^14^,^15^. For example, biocidal single-walled carbon nanotubes (SWNTs) and graphene oxide (GO) nanosheets were used to functionalize polyamide membranes by Elimelech’s group^13^,^15^, which show enhanced antibacterial activity. However, these 1 D or 2 D carbon materials on membranes still cannot be highly efficient in inhibiting the colonization of bacteria.

Graphene oxide quantum dots (GOQDs) with the diameter in the range of 3–20 nm^16^, have attracted extensive attention in the antibacterial research area due to their unique properties. Compared with bulk GO nanosheets, GOQDs exhibit zero-dimensional (0 D) distinctive electronic and optical properties owing to their large edge effects and quantum confinement. This results in better peroxidase-like activity than graphene nanosheets because of their unique electron transport property^17^,^18^,^19^,^20^. More importantly, previous studies have shown that 0 D GOQDs also exhibit lower cytotoxicity than that of the micrometer-sized graphene oxide sheets in a long-term *in vivo* study^21^,^22^. However, to the best of our knowledge, GOQDs have rarely been explored as bactericidal reagents in membranes. The fabrication of antimicrobial, mechanically strong and high-permeability membranes using 2 D graphene sheets incorporated into polymer membrane through mixed-solution casting method has been reported^23^,^24^. However, the solution-casting method may not be preferable, since it leads to the embedment of most graphene materials, inside the membrane, and only a small fraction of graphene is exposed on the surface to exert their functions^15^. Therefore, in order to fully utilize the surface-anchored graphene so as to enable the direct contact with bacteria and significantly reduce the amounts of nanomaterials required, it is highly desirable to homogenously immobilize graphene-based materials on the membrane surface.

In this work, we report a new type of GOQDs covalent-surface functionalized PVDF membrane possessing attractive combined features of hydrophilicity, stability, antibiofouling, and antibacterial properties. Structural analysis, hydrophilicity, water permeability, antibiofouling, and antibacterial performances of the membranes were assessed for its preliminary suitability in water purification processes. It was found that GOQDs anchored PVDF membrane demonstrated significantly-improved antibacterial and antibiofouling performances, while retaining the permeating properties of pristine PVDF membrane. Its antibacterial activity is also superior to previously reported 2 D GO sheets and 1 D carbon nanotube modified polymer membranes^12^,^13^,^14^,^15^.

## Results and Discussion

**Figure 1:**
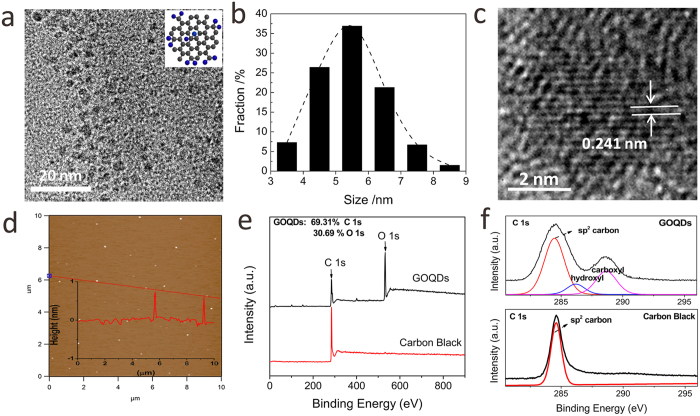
(**a**) TEM image and (**b**) Size distribution of GOQDs; (**c**) High-resolution TEM (HRTEM) and (**d**) AFM images of GOQDs, inset shows the height profile along the red line; (**e**) Survey and (**f**) high-resolution XPS spectra of C 1s for GOQDs and carbon black.

GOQDs were fabricated from carbon black via a hydro-thermal oxidation approach^25^. Typically, XC-72 carbon black was refluxed in a concentrated HNO_3_ solution (65 wt%) at 110 °C for about 24 h. After cooling to room temperature, the mixture was diluted with plenty of deionized (DI) water and then filtered with a 10 kDa molecular weight cut-off membrane. The percolate was subsequently dried in oven at 60 °C. The as-obtained GOQD sample was characterized by transmission electron microscopy (TEM). As can be seen from Fig. 1a,b, the as-prepared GOQDs exhibit an average diameter of ~5.5 nm with a relatively narrow size distribution. A high-resolution TEM (HRTEM) image (Fig. 1c) indicates that GOQDs possess good crystallinity with a lattice spacing of 0.241 nm, which corresponds to (1120) lattice fringe of graphene. Furthermore, atomic force microscopy (AFM) image reveals that the topographic height of GOQDs is about 0.7 nm (Fig. 1d), which is consistent with the thickness of monolayer graphene. The GOQD sample is further investigated by X-ray photoelectron spectroscopy (XPS) and Fourier transform infrared spectroscopy (FTIR). As observed in Fig. 1e, XPS spectrum of GOQD reveals a strong O signal compared to that of carbon black, suggesting the presence of abundant oxygen-containing functional groups in GOQDs after acid-oxidation treatment. Based on the quantitative analysis of the XPS spectrum, atomic ratios of C 1 s to O 1 s in GOQDs and GO were determined to be 2.26 and 2.18, respectively (Figs 1e, S2b), which is consistent with the reported values of graphene related materials^26^. For the high-resolution C 1 s spectrum of the GOQD, it presents two additional signals at 286.2 and 288.6 eV, respectively, corresponding to hydroxyl groups and carboxyl groups (Fig. 1f). The presence of oxygen-containing functional groups, including carboxyl, carbonyl and hydroxyl groups, was further confirmed by the FTIR spectrum (Supporting information Figure S1a). Raman spectrum exhibits two characteristic signals, which are attributed to D band at 1359 cm^−1^ and G band at 1599 cm^−1^ (Figure S1b) of the GOQDs. UV-vis absorption spectrum shows that GOQDs aqueous solution possess a broad absorption below 580 nm, and the emission wavelengths are excitation-dependent and exhibit a red shift with increasing excitation wavelengths, which might be due to the different emissive sites of the synthesized GOQDs (Figure S1c).

**Figure 2:**
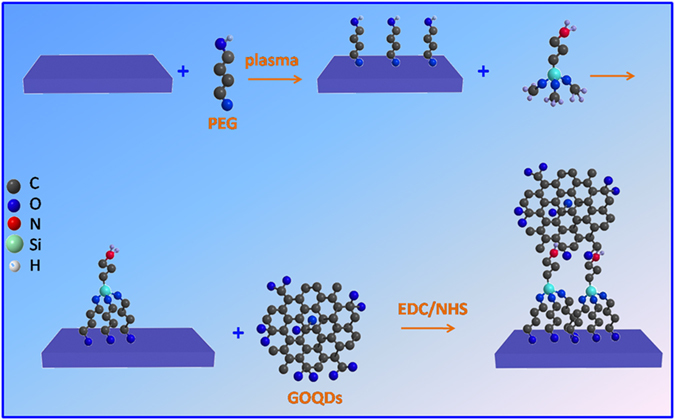
Schematic illustration for covalent immobilization of GOQDs onto the PVDF membrane surface.

The synthetic procedure for GOQDs-functionalized PVDF (GOQDs-PVDF) membrane was depicted in Fig. 2. The PVDF membrane surface was firstly hydroxylated via helium plasma induced grafting of polyethylene glycol (PEG). The PEG-grafted PVDF membrane surface was then treated with 3-aminopropyl-trimethoxysilane (APTMS) to introduce amine groups, which allows for covalent linkage of GOQDs through the formation of covalent bonds between the carboxylic groups on GOQDs and the amine groups on the PVDF surface in the presence of ethyl(dimethylaminopropyl) carbodiimide/N-hydroxysuccinimide (EDC/NHS), thus leading to the formation of GOQDs-PVDF membrane.

**Figure 3:**
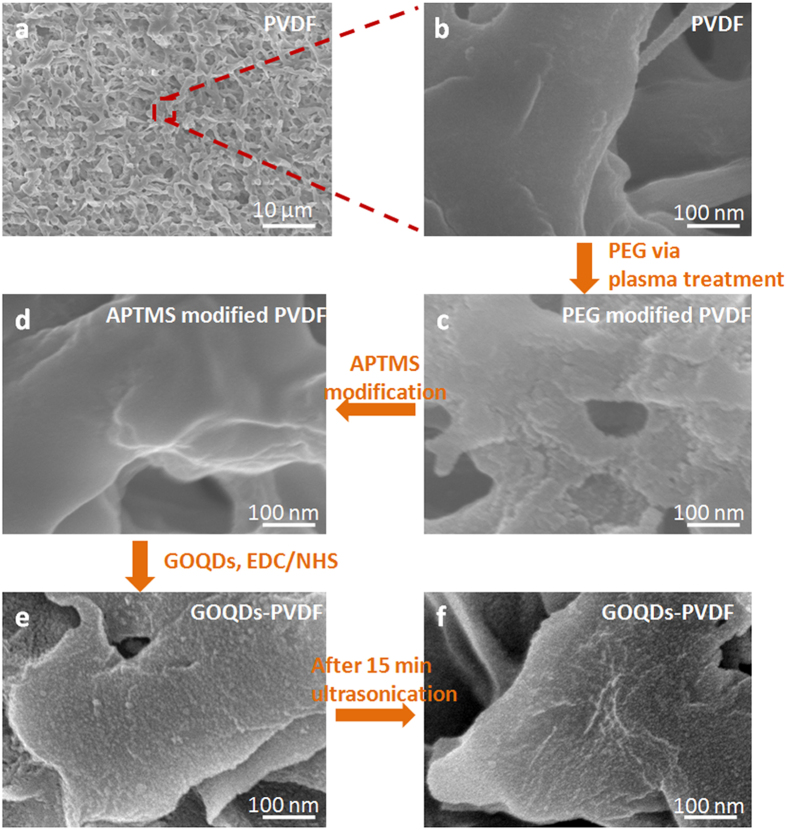
SEM images of (**a,b**) pristine PVDF, (**c**) PEG modified PVDF, (**d**) APTMS modified PVDF, (**e**) GOQDs-PVDF and (**f**) GOQDs-PVDF membranes after 15 min ultrasonication treatment.

The original and functionalized PVDF membranes with an average pore size of 0.22 μm were characterized by scanning electron microscopy (SEM). It is evident that original PVDF membrane displays a porous structure with a smooth interior surface (Fig. 3a,b). In contrast, after helium plasma treatment with PEG, the interior surface for the plasma-treated membrane becomes rough, as shown in Fig. 3c, while the membrane after the subsequent modification of APTMS remains smooth (Fig. 3d), similar with that of original PVDF membrane. Contrary to the smooth surface morphology in Fig. 3d,e shows the uniform coating of nanoparticles on the PVDF surface, suggesting the successful immobilization of GOQDs. Notably, GOQD-PVDF membrane still retains a good structural integrity even after ultrasonication treatment for 15 min. The good stability should result from the strong covalent linkage formed between the abundant carboxyl groups on GOQDs and the amino groups of APTMS-modified PVDF membrane.

**Figure 4:**
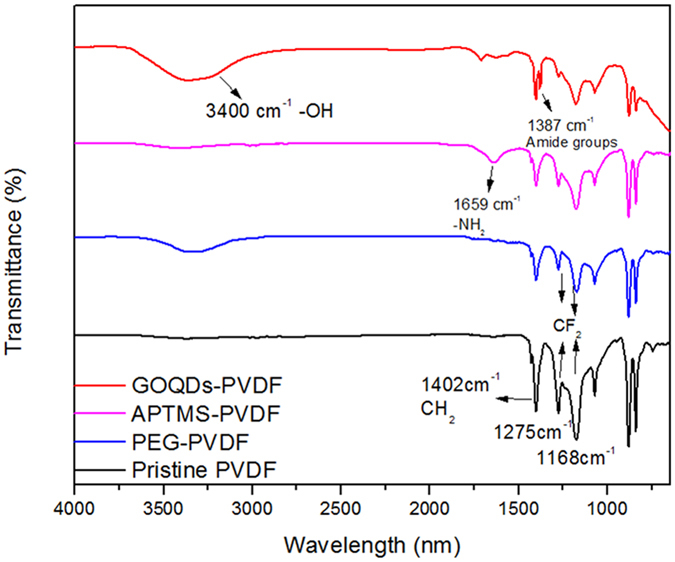
ATR-FTIR spectra of pristine PVDF, PEG modified PVDF, APTMS modified PVDF and GOQDs-PVDF membranes.

The covalent immobilization of GOQDs on the PVDF membrane surface is further evidenced by ATR-FTIR spectra (Fig. 4). As can be seen, original PVDF membrane shows a strong band at 1402 cm^−1^ corresponding to the stretching vibration of C-H bonds, while the bands located at 1275 cm^−1^ and 1178 cm^−1^ are attributed to the vibration of C-F bonds. Compared with the spectrum of pristine PVDF membrane, the asymmetrical and symmetrical stretching of C-F bonds (1178 cm^−1^ and 1275 cm^−1^, respectively) and the vibration of C-H bond (1042 cm^−1^) became weaken obviously after PEG grafting modification, which is due to the dehydrofluorination. The adsorption band at around 3400 cm^−1^ is assigned to hydroxyl groups stretching, which was clearly observed after modification. It is proposed that dehydrogenation of PEG and dehydrofluorination of PVDF under high-vacuum helium plasma environment produced radicals, which subsequently reacted to of hydroxyl groups on the PVDF membrane surface^27^,^28^. This result confirms a successful PEG grafting onto the PVDF membrane surface. Compared with pristine PVDF membrane, a new band at 1659 cm^−1^ appears in the FTIR spectrum of APTMS-modified PVDF membrane, assigned to the stretching vibration of -NH_2_. This result confirms the effective grafting of APTMS on the PVDF surface. After GOQDs immobilization, the signal of -NH_2_ band at 1659 cm^−1^ is attenuated and a new band is observed at 1387 cm^−1^ in the FTIR spectrum of GOQDs-PVDF, which is attributed to the stretching vibration of amide bonds (-NH-CO-). This suggests the presence of covalent linkage of GOQDs with the PVDF membrane. Furthermore, the strong band at 3400 cm^−1^ should arise from the -OH groups of GOQDs. It can also be found that Raman spectra of GOQDs-PVDF and GO-PVDF membranes exhibit the obvious G band (1596 cm^−1^) and D band (1347 cm^−1^) of graphene structure (Figure S5). Therefore, FTIR and Raman results, in conjunction with the above SEM observation, jointly confirm the covalent decoration of GOQDs on the PVDF membrane. Through a zeta potential test, APTMS-modified PVDF membrane was found to have an isoelectric point of 9.8 and was positively charged due to the protonation of amine group. However, the deprotonation of carboxyl group results in GOQDs-PVDF or GO-PVDF membrane to be negatively charged in a wide pH range from 3.8 to 10.2 (Figure S3).

**Figure 5:**
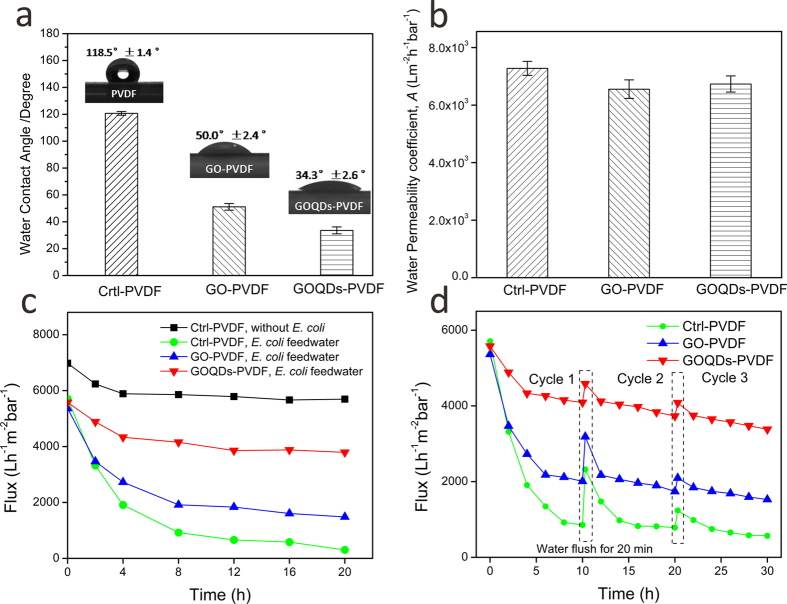
Membrane properties of pristine PVDF, GO-PVDF and GOQDs-PVDF membranes. (**a**) Water contact angle. (**b**) Water permeability coefficient, A. (**c**) The flux change using *E. coli*-containing feedwater during a 20 hr continuous filtration test. (**d**) The flux of PVDF membrane, GO-PVDF membrane and GOQDs-PVDF membrane with three cycles of *E. coli*-containing solution filtration. Before multi-cycle filtration operations, the membranes were forward flushed using pure water for 20 min, and then the feedwater was changed to use *E. coli* solution.

Membrane propensity for antibiofouling depends on the physicochemical properties of the membrane surface, and a key property is the hydrophilicity of the surface. The hydrophilicity of the membrane surface can be evaluated by measuring the water contact angle (CA). The natural wettability of the membrane surface can be reflected by the CA parameter measured immediately through water droplets on the material surface^29^. The pristine PVDF membrane shows a relatively hydrophobic surface with a CA of 118.5°. After modified with 0 D GOQDs, CA value decreases dramatically to 34.3°, which is even smaller than that of 2 D GO nanosheet (Figure S2) modified PVDF (GO-PVDF) membrane (50.0°) produced by similar synthetic procedure (Fig. 5a). The significantly-improved hydrophilicity of GOQDs-PVDF membrane should be due to the presence of abundant oxygen-containing functional groups on GOQDs which are evenly distributed on the membrane surface. Figure 5b presents the flux test results of pristine PVDF membrane, GO-PVDF membrane, and GOQDs-PVDF membrane. It is observed that after modification of 0 D GOQDs sheets, pure water permeability coefficient decreases slightly to (6.83 ± 0.28) × 10^3^ L m^−2^ h^−1^ bar^−1^, relative to that of pristine PVDF membrane, (7.07 ± 0.24) × 10^3^ L m^−2^ h^−1^ bar^−1^, while the decrease in flux is lower than that of 2 D GO nanosheets modified PVDF membrane ((6.55 ± 0.32) × 10^3^ L m^−2^ h^−1^ bar^−1^). This is attributed to the uniform surface coverage of small-sized GOQDs on the membrane surface in comparison with large-sized 2 D GO nanosheets (Figure S4).

Bactericidal and antibiofouling properties of GOQDs-PVDF and GO-PVDF membrane were investigated by using *Escherichia coli* (*E. coli*) as a model bacterium which are similarly described in existing studies^5^,^10^. *E. coli*-containing feedwater was made from a neutral mixture solution of HEPES/glucose (pH 7.0), which can afford a nutritious environment to keep the bacteria survived during long-time filtration. Taking into account the effect of the medium on permeation, a control flux measurement was carried out for a blank PVDF membrane using HEPES/glucose solution as a feedwater. Figure 5c shows a flux drop of about 15.6% during the initial 4 h and a subsequent 3.2% drop after the next 16 h. The initial decrease might be caused by the compaction of medium and the permeation of ionic species into the pores of PVDF. This implies that the medium does not affect the hydrodynamics of the membrane. In contrast, after adding *E. coli* to the feedwater (10^6^ CFU), the resulting solution can significantly affect the permeation of PVDF membrane. As observed in Figs 5c, S7, the flux of pristine PVDF decreases 88.4% after 12 h filtration, suggesting that PVDF membrane can be easily biofouled. In contrast, the permeation decrease of PEG-modified PVDF, APTMS-modified PVDF and GO- PVDF membranes were 79.0%, 76.2% and 65.7%, respectively, while the flux drop of GOQDs-PVDF membrane was only 24.3% over the same duration of filtration. These results strongly suggest that the GOQDs-PVDF membrane possesses a much better biofouling resistance compared to pristine PVDF, PEG-modified PVDF, APTMS-modified PVDF and GO-PVDF membranes. More importantly, the permeation value of GOQDs-PVDF remains stable even after 10 h. During a multi-cycle operation of the filtration with *E. coli* solution (Fig. 5d), the final flux of GOQDs-PVDF membrane is two times larger than that of GO-PVDF membrane, although the performances of both composite membranes can be largely-maintained after three filtration cycles. It is also observed that GOQDs-PVDF membrane can be easily cleaned and the flux can be recovered after forward flushing using DI water. However, the permeability of pristine PVDF membrane showed a significant decrease, and, it cannot be recovered to the initial value after washed by DI water. This result indicates that the fouling resistance of membrane can be significantly improved by the introduction of a uniform GOQDs layer which consists of abundant oxygen-containing functional groups on the membrane surface^30^. In spite of this, the current modified membrane is highly enhanced towards biofouling resistance with the potential of antibiofouling, but will require more extensive studies in order to prove its true antibiofouling capability.

To provide deeper insight into the antibiofouling behavior of the functionalized membrane, the interaction between bacteria and membrane was further investigated. The proliferation activities of *E. coli* and *Staphylococcus aureus* (*S. aureus*) on the pristine and modified PVDF membranes were measured via a plate counting method. After attached to GOQDs-PVDF membrane for 1 h, the metabolic activity of *E. coli* cells was reduced by 88.9% (Figure S8), while the decrease of metabolic activity of *E. coli* on the 2 D GO modified PVDF membrane was 69.2% over the same period of time (1 h), similar with a previously reported GO functionalized polyamide membrane^15^. Alternatively, *S. aureus* cells colonies were exposed simultaneously to pristine PVDF, GO-PVDF and GOQDs-PVDF for 1 h, which showed a 77.9% and 53.7% decrease of metabolic activity of *S. aureus* cells on GOQDs-PVDF and GO-PVDF membranes, respectively (Figure S9). In addition, bacterial inactivation of the current 0 D GOQDs modified PVDF membrane is observed to be more effective than that of 1 D SWNTs anchored polyamide membrane, which shows 60% inactivation of bacteria in 1 h^13^. Thus, the superior antimicrobial activity of GOQDs-PVDF relative to GO-PVDF should be attributed to the uniform dispersion of GOQDs with a larger fraction of active edges on the membrane surface, enabling direct contact with and/or physical piercing to bacterial cells.

**Figure 6:**
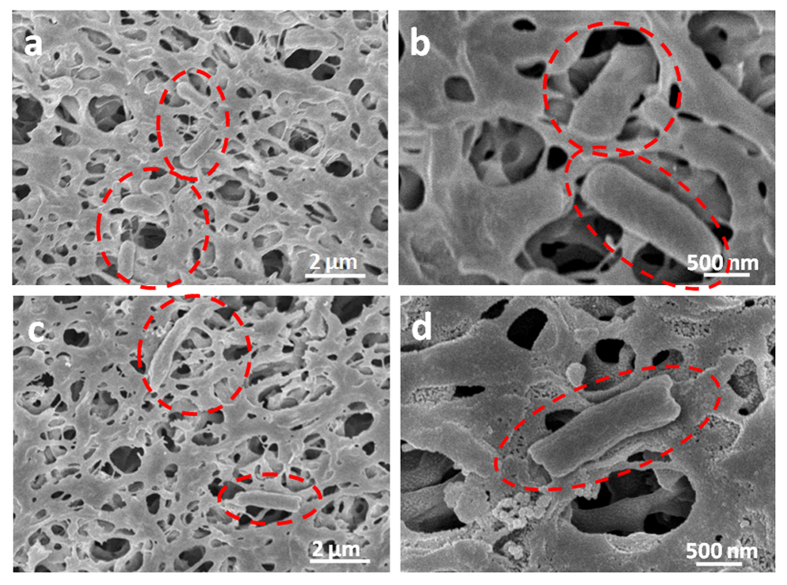
SEM images for normal *E.coli* cells at the surface of pristine PVDF membrane (**a,b**) and GOQDs-PVDF membrane (**c**,**d**).

**Figure 7:**
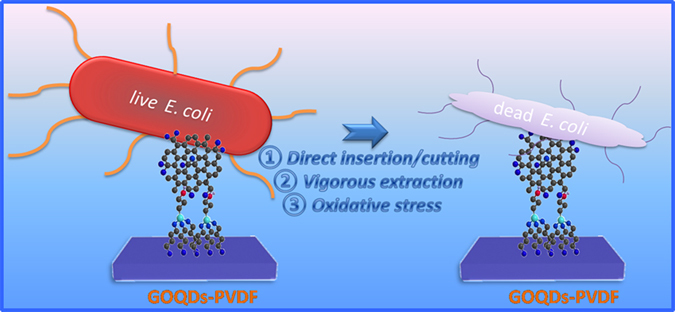
Schematic illustration for the bactericidal mechanism of GOQDs-PVDF.

In order to elucidate the antibacterial mechanism of GOQDs-PVDF membrane, SEM observation was used to provide visual information of the bacterial content on the membrane surface. Most of *E. coli* cells on the GOQDs-PVDF membrane surface have compromised structure (Fig. 6c,d), while the cells attached to pristine PVDF membrane maintain the original structure (Fig. 6a,b). Some *E. coli* cells in contact with antibacterial GOQDs become flattened or shrunken, while other bacteria in a binary fission process grow elongated^31^. In previous studies, the proposed graphene-based cytotoxicity mechanism mainly includes membrane stress induced by direct physical interaction and oxidative stress^14^,^32^. In our case, we suggest that the highly-dispersed GOQDs with the ultra-small size and large specific surface area, similar to those of the graphene nanosheets reported by Akhavan *et al*.^30^ and Tu *et al*.^32^, can perform a contact-inhibition mode, which is beneficial for direct insertion/cutting of lipid membranes and vigorous extraction of phospholipid molecules due to the strong dragging forces from graphene’s unique structure of sp^2^ carbons, thus resulting in severe destruction of cell membrane and eventual loss of cytoplasm (Figs 6c,d and S10). The lipid extraction in the outer and inner membrane of *E. coli* can be induced by the strong dispersion interactions between GOQDs and lipid molecules^32^. Even for bacterial cells in motion, as reported in the works involving polyacrylonitrile (PAN) hollow fiber decorated with silver nanoparticle/multiwalled carbon nanotube (Ag/MWNTs)^5^ and the grafting of polyethyleneimine-coated silver nanoparticles on the surface of a plasma-treated polysulfone membrane^33^, the immobilization of nanomaterial into appropriate matrixes could offer a potential solution to the development of a disinfection system with good reliability and ease of operation. Similarly, GO nanosheets decorated membrane can also have bactericidal activity with a similar mechanism as described above on *E. coli* cells. However, due to their relatively tumid lateral size and wider size distribution, they could not fully cover the membrane surface with high dispersion (Figure S4). Some area without GO were exposed, enabling bacterial colonization and subsequently leading to the formation of cohesive biofilm structures. Therefore, GOQDs-PVDF membrane is more effective in inhibiting the colonization of bacteria and the biofilm formation in comparison with pristine PVDF and GO-PVDF as depicted in Fig. 7.

Oxidative stress is another way of inducing cell death, in which a specific vital cellular component or structure was oxidized or disrupted by graphene-based materials^14^. Direct contact of bacteria with GO edges may not necessarily lead to bacterial inactivation^34^,^35^. Herein, glutathione (GSH) oxidation *in vitro* is further employed to evaluate the oxidative stress induced by GO or GOQDs. GSH, a tripeptide molecule with thiol groups, can protect cellular components from the oxidative stress, is converted to glutathione disulfide when the thiol groups are oxidized to disulfide bond. The concentration of thiol groups of GHS can be quantified by the Ellman’s assay, and the oxidative stress can be evaluated by monitoring the ratio of GSH loss^14^. The oxidation of GSH was evaluated during the incubation with pristine PVDF, GO-PVDF, and GOQDs-PVDF. As shown in Figure S11, a noticeable fraction of GSH is oxidized after its exposure to GOQDs-PVDF (63.3 ± 2.42%) and GO-PVDF (22.1 ± 2.67%). Several studies have shown that GSH oxidation for single walled carbon nanotubes (SWNTs) and graphene-based materials is attributed to their excellent electronic conductivity^13^,^14^. When carbon nanomaterials are highly dispersed on the PVDF membrane surface, the facilitation of carbon nanomaterial networks are indicated as a unique combination of the electrons transport^36^. In our cases, we suggest that the difference of GSH oxidation extent between GOQDs-PVDF and GO-PVDF arises from the structure difference between 0 D GOQDs and 2 D GO. The homogeneous dispersion of smaller GOQDs on the same membrane surface, relative to GO sheets with lateral dimension, could lead to the exposure of a much larger fraction of active edges, facilitating the formation of a higher oxidative stress. Nonetheless, the bactericidal activity of the current GOQDs functionalized membrane, such as the exact contribution of physical or oxidative stress, and detailed oxidative pathways, can be further investigated in future studies.

**Figure 8:**
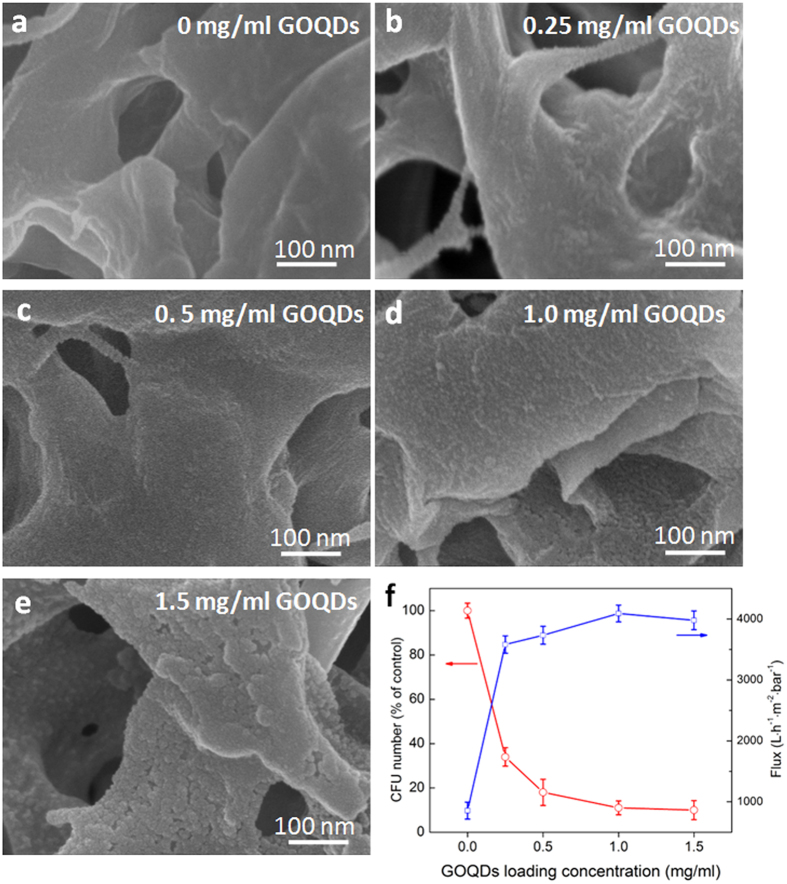
(**a**–**e**) SEM images of GOQDs-PVDF membranes with different loadings amount of GOQDs, which are prepared by changing the concentration of GOQDs in the aqueous solution. (**f**) The loss number of CFU (after 1 h contact) and the flux change of GOQDs-PVDF (after 10 h filtration) with different loading amount of GOQDs.

By tuning of GOQDs concentration in the precursor solution, the effect of the GOQDs loading on the PVDF membranes on the antibacterial and the permeation performances were also studied. As shown in Fig. 8, the loading amount of GOQDs on the surface of membrane increases gradually with the increase of the concentration of aqueous GOQDs solution, and a full coverage of GOQDs on the surface was achieved at a concentration of ~1.0 mg/ml. Using the same plate counting method, the proliferation activity of *E. coli* on the composite membranes with different densities of GOQDs was measured. The CFU numbers of bacterial cells decrease with increasing the concentration of GOQDs. Simultaneously, the final flux of the GOQDs functionalized membranes after 10 h permeation test with bacterium-containing feedwater were significantly increased when increasing the concentration of GOQDs. It is noted that the antibacterial activity and biofouling resistance of GOQDs-PVDF membrane were enhanced with increasing the concentration of GOQDs from 0 to 1 mg/ml. When the GOQDs concentration was further increased, the membrane surface exhibited the formation of a multi-layer of particles due to the aggregation of excess GOQDs, and the antimicrobial activity and biofouling resistance showed no significant enhancement (Fig. 8f), which can be attributed to the agglomeration of GOQDs on the membrane surface. The results indicate that the loading amount of GOQDs on the membrane surface should be well controlled to achieve the optimal water permeation, antimicrobial and antibiofouling activity of composite membranes.

## Conclusions

The present study reported a first GOQDs covalent-functionalized PVDF membrane, and explored its use as an antimicrobial agent and preliminary as an antibiofouling membrane. The GOQDs-PVDF membrane shows much improved hydrophilic, antibacterial and antibiofouling properties without sacrificing the permeation property of pristine PVDF membrane. A continuous filtration test using *E. coli*-containing feed water indicated that the flux drop over the GOQD modified PVDF was 24.3%, which is significantly lower than those over pristine PVDF (88.4%) and over the GO-sheet modified PVDF (65.7%) at 12 h of filtration. Moreover, the presence of the GOQD coating layer effectively inhibits the growth of the bacterial cells and prevents the formation of the biofilm on the membrane surface, yielding a higher bacterial inactivation efficiency than those over 1 D SWNTs and 2 D GO modified membrane. The loading of GOQDs on the membrane could be easily tuned to achieve an optimal functionalized membrane combining excellent water permeation property, antimicrobial activity and biofouling resistance. The attractive features of hydrophilicity, stability, antibiofouling, and antibacterial properties of GOQDs-PVDF membrane are promising in further enhancing membrane performance for the desalination/purification of water, and potentially separation of biomolecules.

## Methods

### Synthesis of graphene oxide quantum dots (GOQDs) and graphene oxide nanosheets (GO)

Graphene oxide quantum dots (GOQDs) were synthesized with CX-72 carbon black via being refluxed in concentrated nitric acid (HNO_3_) solution^25^. Typically, 0.4 g CX-72 carbon black was added to 6 mol L^−1^ nitric acid (100 ml) and refluxed for 24 h at 110 °C. After cooling to below 30 °C, the mixture was diluted with plenty of deionized (DI) water and then filtered with a 10 kDa molecular weight cut-off membrane. The percolate was subsequently dried in oven at 60 °C. After cooling to the room temperature, a reddish-brown solid was acquired. Then, GOQDs solution can be obtained by dissolving the GOQDs in DI water under 10 min sonication. Graphene oxide nanosheets (GO, Figure S2) were fabricated via the modified Hummers’ method^37^,^38^.

### Fabrication of GO-PVDF/GOQDs-PVDF

PEG (15 g) was put into DI water (300 g) to prepare a PEG solution (5 wt%). PVDF membrane (pore size 0.22 μm, GVHP from Millipore) was immersed into the PEG solution for 30 min, and then dried in the air. The pretreated membrane was dropped between the biased electrode and ground electrode in a plasma chamber. Helium (He) gas was subsequently channeled into the chamber when the pressure of the reactor system was 0–100 Pa. The radio-frequency power of discharging He plasma was 100 W, while the exposure time was 120 s. To remove the residual PEG, the grafted membrane was washed by DI water for 2 h. The resulting membrane was dried at 60 °C in a vacuum oven for further characterization and preparation.

The resulting membrane was dipped into 0.2 wt% (3-aminopropy) trimethoxysilane (APTMS) in iso-propanol (IPA) for 30 min. The membrane was subsequently rinsed and immersed into DI water for 10 min, and the washing process was repeated for 3 times. After that, the membrane was dried under nitrogen flow. The obtained membrane was then dipped into an aqueous solution of GO, or GOQDs (1 mg/ml) with ethyl(dimethylaminopropyl) carbodiimide/n-hydroxysuccinimide (EDC/NHS) for 3 h. Then, the membrane was again rinsed and immersed by DI water to remove the residual GO. The resulting GO-PVDF or GOQDs-PVDF was dried with nitrogen flow.

### Antimicrobial activities assay

The antimicrobial activity of the composite films was evaluated by a plate counting method. Before counting a functionalized filter for antimicrobial activities, the presence of graphene on the filter was confirmed by performing water contact angle measurements for each filter. Both *E. coli* and *S. aureus* bacteria were prepared in Luria-Bertani (LB) medium at 37 °C for overnight with shaking, and then washed three times with fresh sterile 0.9% saline. The bacteria solution was diluted to ~10^7^ colony-forming units per milliliter (CFU mL^−1^) by using sterile 0.9% saline solution. All the solutions and samples (except the composite films) in the experiment need to be autoclaved at 120 °C for about 20 min to ensure sterility. Antibacterial activities were explored with the antibacterial drop-test method^15^. For composite film exposure, pristine PVDF and functionalized membranes with an area of 2 cm^2^ are placed in a plastic holder to expose the active surface to the bacteria. Bacterial suspensions (0.5 mL per cm^2^) were contacted with pristine PVDF and the functionalized membranes for 60 min at room temperature. The excess bacterial suspension was discarded. The membranes were washed entirely by using sterile 0.9% saline. The functionalized membranes were put into a 50 mL tube with 10 mL of sterile 0.9% saline and ultrasonicated for 10 min to remove the bacteria cells from the membrane^15^. After that, bacteria cells were put on LB agar plates and incubated for 12 h at 37 °C.

To obtain the SEM images of bacterial on the functionalized membranes surface, *E. coli* suspensions were exposed to the membranes as above. After contacting the cells with the functionalized membranes, the membranes were washed by using sterile 0.9% saline solution and fixed with Karnovsly’s fixative (2.5 wt% glutaraldehyde in 0.2 M PBS buffer) for 3 h. Samples were then dehydrated by a sequential immersion in water/ethanol (30, 50, 60, 70, 80, 90, 100%), and left to dry for 12 h in a desiccators below 30 °C. After that, samples need to be sputter-coated by using platinum and were imaged by SEM.

### Filtration module setup and permeation test

The membrane was fixed on the flat sheet membrane (5 cm × 5 cm, effective flow area is 5.25 cm^2^). The filtration module was set up as shown in Figure S6. The membrane was operated with cross-flow mode, during which the flow rate was fixed at 1.0 L/min. DI water was permeated through the membrane module for 30 min for initial flux measurement. Transmembrane pressure (TMP) was monitored by a digital pressure gauge, which was fixed at ~1 bar. The permeation test for pristine PVDF, GO-PVDF and GOQDs-PVDF membranes was operated under the same conditions.

### Filtration test of GOQDs-PVDF membrane using bacterial feedwater

The *E. coli* bacterium was cultured in Luria-Bertani broth (5 g/l of yeast extract, 10 g/l of tryptone and 10 g/l NaCl) at 37 °C for 24 h. About 10^9^ colony forming units (CFU) per ml can be obtained. The bacterial cells were separated from the suspension by centrifugation, washed and resuspended in a composite solution of glucose (1 wt %) and HEPES buffer (10 mM), and the final concentration of cells as the feedwater is approximately 10^6^ CFU. Before the multi-cycle operations, the membranes were flushed using pure water for 20 min, then the feedwater was changed to *E. coli* solution. The filtration test for pristine PVDF, GO-PVDF and GOQDs-PVDF membranes was operated under the same conditions (1.0 L/min of flux rate, 1 bar of permeation pressure). Before examining the membrane by FESEM, the samples were performed in freeze dryer at −55 °C for 12 h.

### Oxidative stress of GOQDs-PVDF/GO-PVDF membrane assay

The concentration of thiols in GSH can be evaluated by the Ellman’s assay^14^. The pristine PVDF, GO-PVDF and GOQDs-PVDF (5 × 5 mm^2^) in 50 mM bicarbonate buffer was mixed into 300 μl of GSH. All samples were tested in triplicate. After incubation for 1 h, 800 μl of 0.05 M Tris-HCl and 20 μl of 5,5′-dithio-bis-(2-nitrobenzoic acid) (DNTB, Sigma-Aldrich) were added into the mixtures. A 300 μl aliquot of solution was then placed in a 24-well plate. By using Benchmark Plus microplate spectrophotometer, the absorbance at 412 nm was monitored. GSH solution without membrane was used as a negative control. GSH oxidization by H_2_O_2_ (1.0 mM) was tested as a positive control.

### Characterization

Scanning electron microscopy (SEM) images can be acquired with field emission scanning electron microscopy (JEOL, JSM-6700F). Transmission electron microscopy (TEM) and high-resolution transmission electron microscopy (HRTEM) images were achieved by using a JEOL model JEM2010 EX instrument at an accelerating voltage of 200 kV. X-ray photo electron spectroscopy (XPS) measurements were collected through ESCALAB 250 photoelectron spectrometer (Thermo Fisher Scientific) at 2.4 × 10^−10^ mbar with a monochromatic Al K*α* X-ray beam (1486.60 eV). Binding energy (BE) of the element was calibrated to the BE of carbon (284.60 eV). UV-vis spectra were recorded by using a Shimadzu UV2501 spectrophotometer. The fluorescence properties were studied using a Fluoromax-4, Horiba Jobin Yvon Spectrofluorometer with a photon-counting detection system to detect fluorescence emission. Sample excitation was obtained by using a diode laser, BWF-2 (980 nm, P_max_ = 1.0 W at 3.0A, B&W TEK Inc.) with a optical fiber (100 μm, core). Fourier Transform Infrared Spectroscopy (FTIR) was conducted in a Digilab FTS 3100 instrument by collecting 45 scans with a resolution of 4 cm^−1^. Raman spectroscopy (Renishaw) was equipped with a 633 nm laser source. Membrane surface charges were measured through using streaming potential method with a SurPASS electrokinetic analyzer (Anton Paar GmbH, Austria)^39^,^40^.

## Additional Information

**How to cite this article**: Zeng, Z. *et al*. Graphene Oxide Quantum Dots Covalently Functionalized PVDF Membrane with Significantly-Enhanced Bactericidal and Antibiofouling Performances. *Sci. Rep.* **6**, 20142; doi: 10.1038/srep20142 (2016).

## Supplementary Material

Supplementary Information
