# Supplementary material for: Graphene Oxide Quantum Dots Covalently Functionalized PVDF Membrane with Significantly-Enhanced Bactericidal and Antibiofouling Performances
Source: Sci Rep. 2016 Feb 2;6:20142. doi: 10.1038/srep20142 (PMC4735848; doi:10.1038/srep20142)
Supplement: Supplementary Information [file srep20142-s1.pdf]

Supplementary Information for

Graphene Oxide Quantum Dots Covalently Functionalized PVDF Membrane with Significantly-Enhanced Bactericidal and Antibiofouling Performance

Zhiping Zeng[1,2], Dingshan Yu[3], Ziming He[2], Jing Liu[2], Fang-Xing Xiao[2], Yan Zhang[2], Rong Wang[1], Dibakar Bhattacharyya[4], Timothy Thatt Yang Tan[2]

[1] Singapore Membrane Technology Center, Nanyang Environment and Water Research Institute, Interdisciplinary Graduate School, Nanyang Technological University, Singapore; [2] School of Chemical and Biomedical Engineering, Nanyang Technological University, Singapore; [3]School of Chemistry and Chemical Engineering, Sun Yat-sen University, Guangzhou, China; [4]Department of Chemical and Materials Engineering, University of Kentucky, Lexington, KY, USA

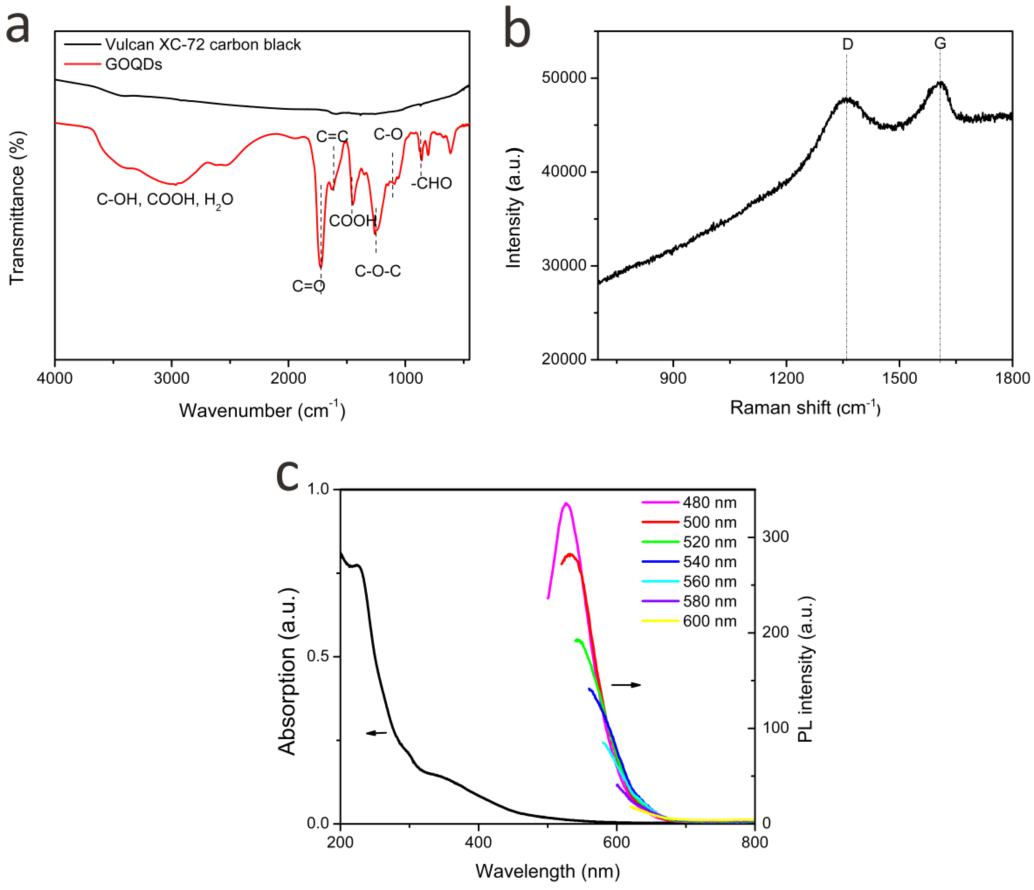

Figure S1. (a) FTIR spectra of carbon black and GOQDs; (b) Raman spectrum of GOQDs; (c) UV-vis absorption and PL emission spectra of the GOQDs aqueous solution.

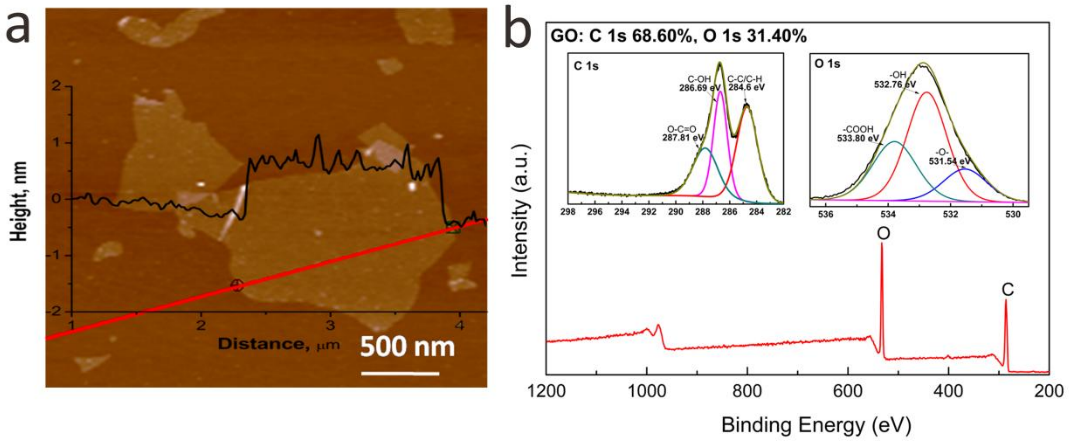

Figure S2. (a) Tapping mode AFM image of GO sheets, inset shows the height profile along the red line; (b) High-resolution XPS spectra of GO.

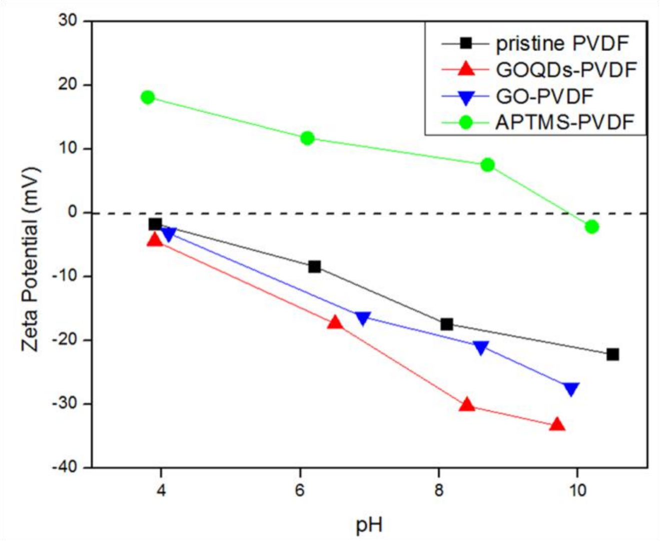

Figure S3. Zeta-potential of pristine PVDF, APTMS modified PVDF, GOQDs-PVDF and GO-PVDF membranes.

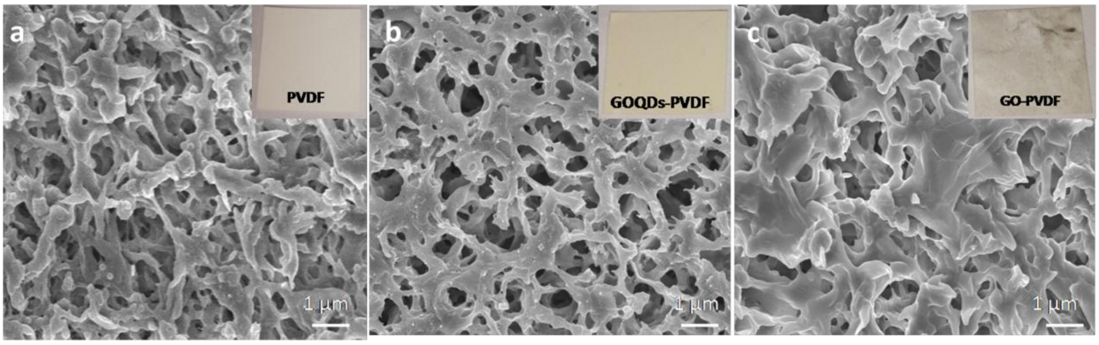

Figure S4. SEM images of PVDF (a), GO-PVDF (b), and GOQDs-PVDF (c). The upper inset are the optical images of the corresponding membranes.

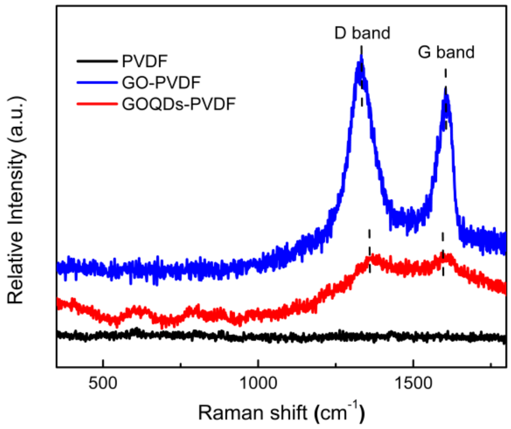

Figure S5. Raman spectra of pristine PVDF, GO-PVDF, GOQDs-PVDF membranes.

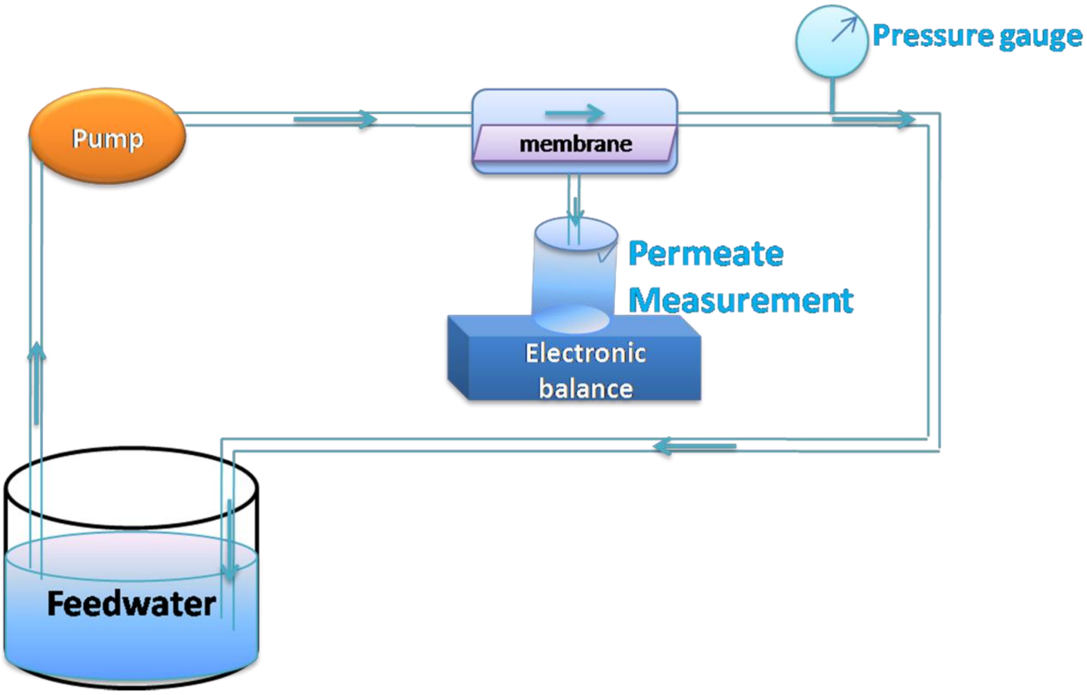

Figure S6. Schematic diagram of the batch experiment setup for filtration test.

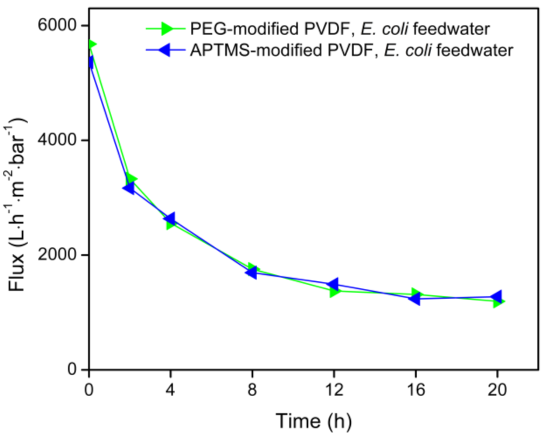

Figure S7. The flux change of PEG-modified PVDF, APTMS-modified PVDF membranes using *E. coli*-containing feedwater during a 20 h continuous filtration test.

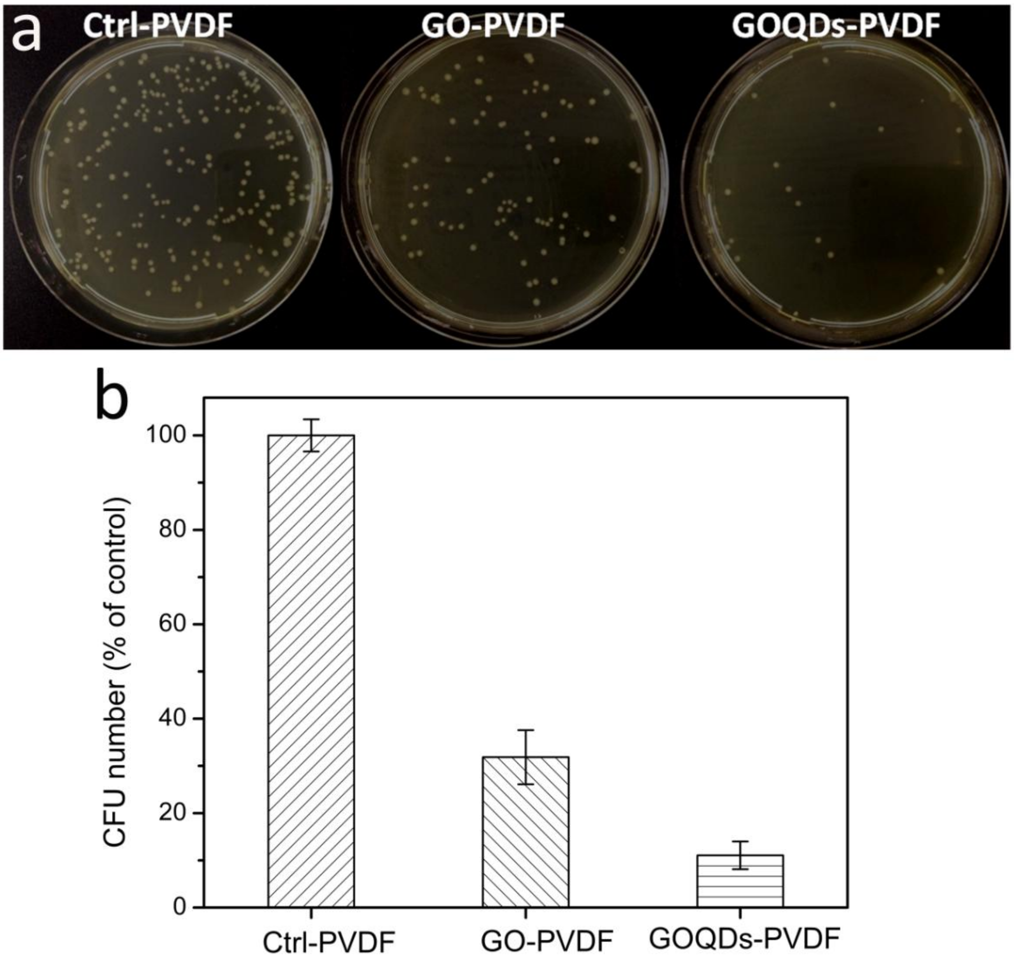

Figure S8. Graphene functionalized membranes reduce the number of *E. coli* cells colonies. (a) *E. coli* suspensions were exposed simultaneously to pristine PVDF, GO-PVDF and GOQDs-PVDF membranes for 1 h. (b) The difference of CFU for the different membranes; CFU was determined by standard plate count method.

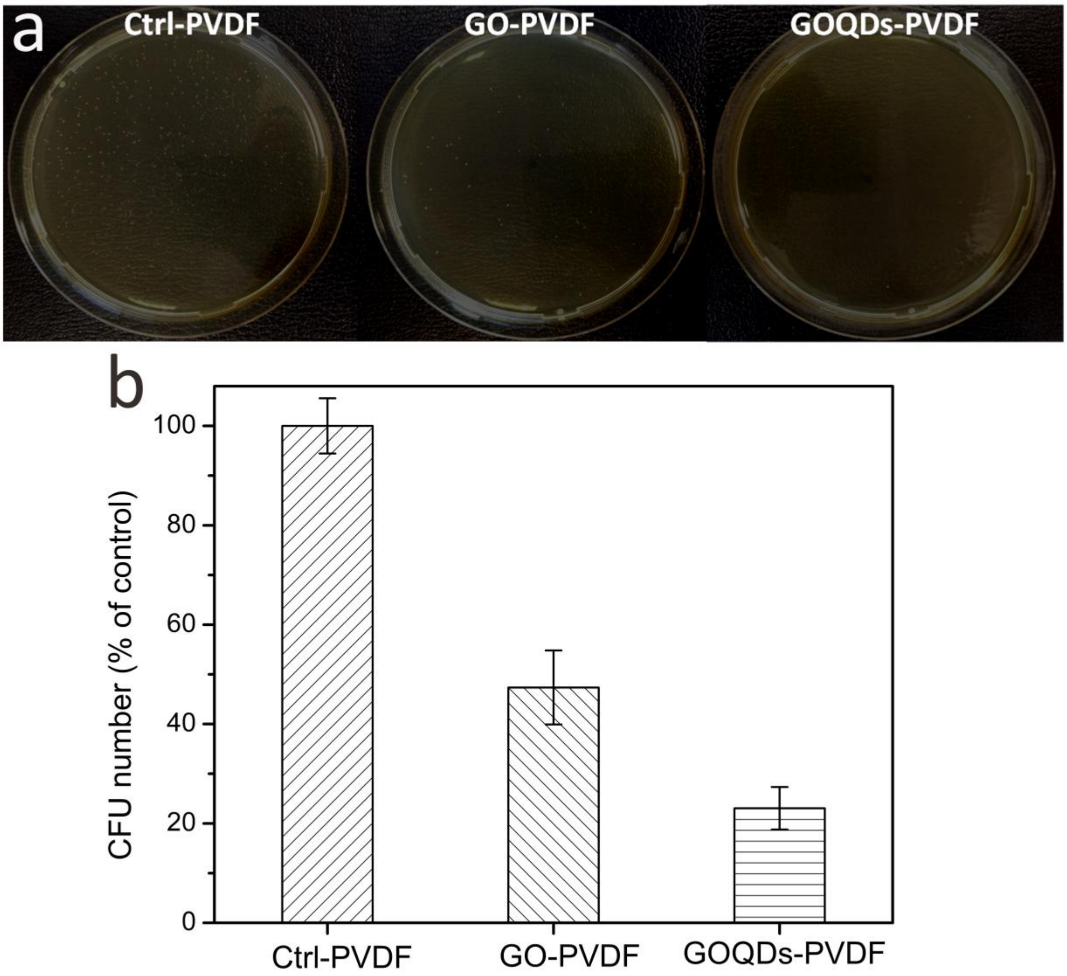

Figure S9. Graphene functionalized membrane reduce the number of *S. aureus* cells colonies. (a) *S. aureus* suspensions were exposed simultaneously to pristine PVDF, GO-PVDF and GOQDs-PVDF membranes for 1 h. (b) The difference of CFU for the different membranes; CFU was determined by standard plate count method.

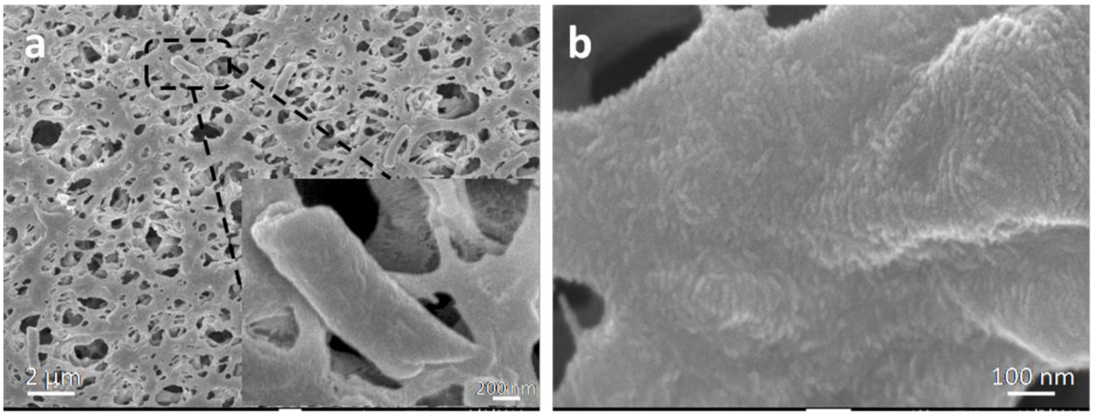

Figure S10. (a) SEM images of GOQDs-PVDF membrane after 10 h filtration test using *E. coli*-containing feedwater; inset the *E. coli* cell on the membrane surface; (b) High-resolution SEM image of GOQDs-PVDF after 10 h filtration test using *E. coli*-containing feedwater.

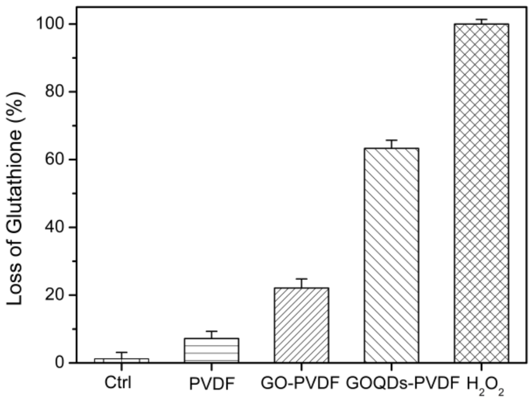

Figure S11. Loss of GSH (0.4 mM) after *in vitro* incubation with pristine PVDF and GO-PVDF for 1 h. H_2O_2 (1 mM) is a positive control. The bicarbonate buffer (50 mM at pH 8.6) without membrane was used as a negative control.
